# Successful Treatment of Multiple Myeloma Complicated With SARS‐CoV‐2 Infection: A Case Report

**DOI:** 10.1155/carm/7967791

**Published:** 2026-02-18

**Authors:** Yuna Meng, Baoying Duan, Lixian Gao, Wenjiao Huang, Fang Wan

**Affiliations:** ^1^ Department of Hematology, The First People’s Hospital of Baiyin, Baiyin, Gansu, China

**Keywords:** case report, infectious disease, multiple myeloma, nirmatrelvir/ritonavir tablets, SARS-CoV-2 infection

## Abstract

**Background:**

Since the declaration of the coronavirus disease 2019 (COVID‐19) pandemic in late 2019, COVID‐19 has remained a global concern. Elderly patients with hematological malignancies are immunocompromised and thus highly susceptible to COVID‐19 infection and severe acute respiratory syndrome (SARS), posing significant clinical challenges. This report describes the diagnosis and treatment of a patient with multiple myeloma (MM) who developed SARS‐CoV‐2 infection.

**Methods:**

We collected general patient information, clinical features, laboratory results, chest CT imaging, electronic bronchoscopy results, and clinical results for analysis. This case report was approved by the Institutional Review Committee of Baiyin First People’s Hospital, and informed consent was obtained from the patient.

**Outcomes:**

We report a case of a 66‐year‐old male newly diagnosed with multiple myeloma who developed a SARS‐CoV‐2 infection following chemotherapy. His clinical condition showed marked improvement after the initiation of nirmatrelvir/ritonavir therapy. This case underscores the heightened susceptibility of immunocompromised patients to SARS‐CoV‐2 and highlights the therapeutic importance of early administration of antiviral agents. A comprehensive understanding of the increased risk of opportunistic infections in patients with multiple myeloma postchemotherapy is essential for optimizing disease management and improving clinical outcomes.

**Conclusion:**

Patients with MM have impaired immune function and are at high risk for severe infections during treatment. Proactive infection prevention and early intervention are essential to improving outcomes during treatment.

## 1. Introduction

Coronavirus disease 2019 (COVID‐19), caused by infection with severe acute respiratory syndrome coronavirus 2 (SARS‐CoV‐2), presents with a wide range of symptoms. Approximately 80% of cases are mild, while about 20% progress to severe illness and 5% develop critical conditions such as pneumonia or acute respiratory distress syndrome (ARDS), often requiring mechanical ventilation and intensive care unit admission [1]. Patients with COVID‐19 who have underlying comorbidities, such as hypertension, diabetes mellitus, and cardiac or renal diseases, are at significantly higher risk for severe complications, including septic shock, ARDS, and even death [2]. Since its emergence in December 2019, COVID‐19 has rapidly spread worldwide, leading the World Health Organization (WHO) to declare a global pandemic on March 11, 2020 [3]. SARS‐CoV‐2 infection can coexist with various malignancies, including multiple myeloma (MM). In such cases, complications such as hyperinflammation, multiorgan dysfunction, and thrombosis may be exacerbated, increasing disease severity. Patients with MM typically have impaired immune function, increasing their susceptibility to severe infections during treatment, which can be life‐threatening. Therefore, active preventive measures and timely clinical interventions are critical. This report presents the clinical management of a patient with MM who was concurrently infected with SARS‐CoV‐2. No written consent has been obtained from the patient as there are no patient identifiable data included in this case report.

## 2. Case Report

**FIGURE 1:**
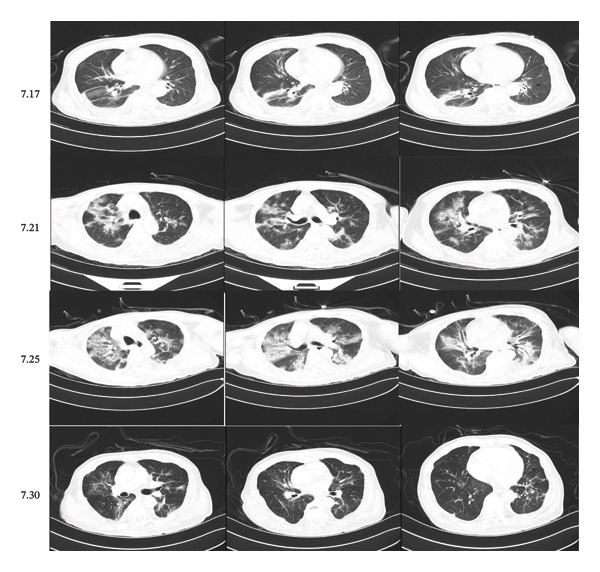
Chest CT changes of patients at different dates.

On July 3, 2023, a 66‐year‐old man was admitted to the hospital due to “intermittent sternum and low back pain for more than 1 month, aggravated for 1 week.” Blood routine results were as follows: hemoglobin (HGB) 101 g/L, platelet count (PLT) 250 × 10^9^/L, red blood cell (RBC) count 3.49 × 10^12^/L, and white blood cell (WBC) count 5.05 × 10^9^/L; total protein (TP) 125.4 g/L, albumin (LB) 22.2 g/L, globulin (GOLB) 103.2 g/L, alanine aminotransferase (ALT) 8 U/L, aspartate aminotransferase (AST) 10 U/L, immunoglobulin G (IgG) 81.62 g/L, and C‐reactive protein (CRP) 62.0 mg/L. Bone marrow cytomorphology demonstrated 21.5% plasma cells, which were medium‐sized with large amounts of cytoplasm appearing blue‐purplish to reddish, pseudopodia, round nuclei, detailed chromatin, and nucleoli visible in some cells. Binuclear and trinuclear plasma cells were easily observed. Flow cytometric immunophenotyping revealed that plasma cells accounted for approximately 2.53% of the total nuclear cells, expressing CD38+, CD138+, CD19−, CD20−, CD28+, CD56−, CD81−, and CD200−. Intracellular immunoglobulin kappa light chain was restricted, suggesting a monoclonal plasma cell population. Serum protein electrophoresis showed an M protein percentage of 58.50% and a serum M protein content of 77.06 g/L. Urine protein electrophoresis showed a urine M protein percentage of 49.4% and a urine M protein content of 421.38 mg/24 h. IF analysis showed that the monoclonal immunoglobulin type was IGg‐κb type. B‐J typing results showed that precipitation bands were found in immunoglobulin G, A, and M, *κ* light chain and *κ* free light chain swim lanes, and urine Bence Jones protein was positive, the type was *κ* free light chain type. Karyotype analysis showed 46,XY [4]. Chest CT showed changes in the sternum, bilateral clavicle, scapulae, ribs, and thoracolumbar vertebrae, with pathological fractures noted in bilateral ribs (Figure 1 (7.17)). Multiple abnormal signals were observed in the thoracolumbar and lumbar vertebrae, vertebral appendages, and bilateral iliac wings, suggestive of MM. Cranial MRI revealed abnormal signals in the bilateral frontal, temporal, occipital, and parietal bones, indicating possible myeloma. Whole‐body bone scan demonstrated increased metabolic activity in multiple ribs, raising the suspicion of fractures. Bone mineral density assessment revealed osteoporosis. The patient was diagnosed with IgG‐κ type MM, Durie–Salmon (DS) Stage III, Subtype A, ISS Stage II, R‐ISS Stage II. After ruling out contraindications, systemic chemotherapy with the VRD (bortezomib + lenalidomide + dexamethasone) regimen was initiated on July 11.

**FIGURE 2:**
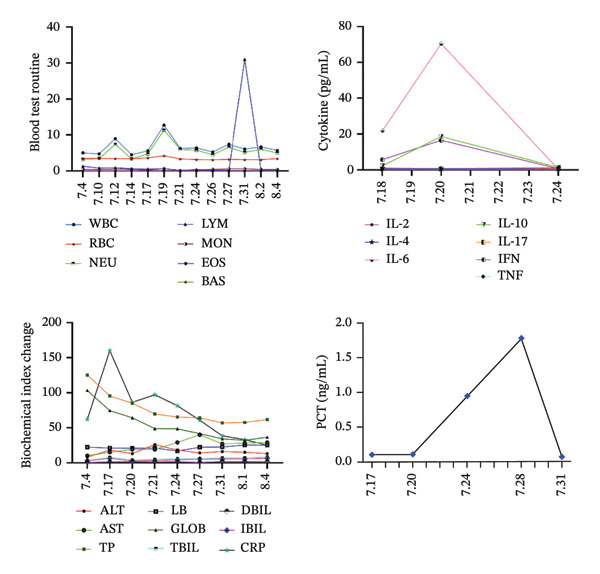
Changes of blood routine, biochemistry, cytokines, and PCT at different dates.

**FIGURE 3:**
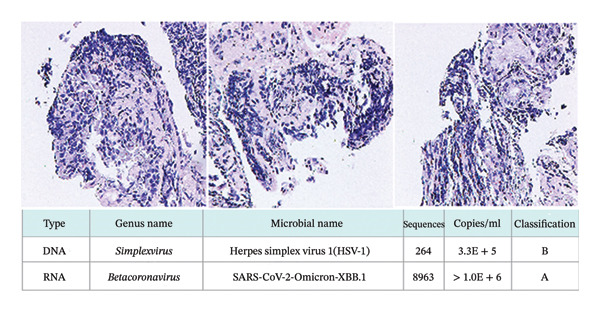
Pathology of lung tissue and detection of SARS‐CoV‐2 infection.

On the 11th day of chemotherapy, the patient developed fever, intermittent cough, and sputum. The trends of blood routine, biochemistry indices, procalcitonin (PCT), and cytokine, including interleukin‐2 (IL‐2), IL‐4, IL‐6, IL‐10, IL‐17, interferon (IFN), and tumor necrosis factor (TNF), are shown in Figure 2. Follow‐up chest CT examination suggested bilateral pneumonia (Figure 1 (7.21)). Based on clinical presentation and imaging findings, the patient was diagnosed with severe pneumonia. Empiric anti‐infective therapy was initiated with intravenous piperacillin sodium and tazobactam sodium (4.5 g every 12 h) for 3 days. However, the patient continued to experience intermittent high‐grade fever with a temperature of up to 40.6 °C, accompanied by chills, chest tightness, dyspnea, paroxysmal coughing, and hemoptysis. The sputum was difficult to expectorate. Inflammatory markers including PCT, CRP, and cytokines were higher than before. Antibiotic therapy was changed to meropenem for anti‐infection treatment, but the patient’s fever persisted, and a self‐administered COVID‐19 antigen test returned positive. Repeat chest CT showed worsening bilateral pneumonia with more localized lesions and increased extent of pulmonary involvement (Figure 1 (7.25)), along with bilateral pleural effusion, right‐sided compression atelectasis, and a bulla in the left lower lobe. Bronchoscopy with bronchoalveolar lavage and lung biopsy was performed under local anesthesia. Next‐generation sequencing (NGS) of the lavage fluid detected high pathogen concentrations of novel coronavirus (Omicron‐XBB.1, > 1 × 1.0^6^) and herpes simplex virus 1 (HSV‐1, 3.3 × 10^5^). Histopathological examination of the lung tissue (the right upper posterior lobe) showed acute and chronic mucosal inflammation and severe focal tissue compression. Immunohistochemical results revealed lymphatic hyperplasia with the following markers: Ki67 nuclear positive index 0%, CKp+, NapsinA−, TTF−1−, Syn−, CgA−, CD56−, and LCA+ (Figure 3). The patient was treated with nirmatrelvir/ritonavir for COVID‐19, methylprednisolone sodium succinate for anti‐inflammation, antiviral effects, and supportive care including prone positioning and high‐flow oxygen therapy. Subsequently, the patient became afebrile, and symptoms of cough and sputum production improved. Follow‐up chest CT examination showed partial absorption and resolution of pulmonary lesions (Figure 1 (7.30)). The English abbreviations and full English names of the research indicators and disease terms mentioned in this study are listed in Table 1.

**TABLE 1:** English abbreviation and full English name.

English abbreviation	Full English name
MM	Multiple myeloma
SARS‐CoV‐2	Severe acute respiratory syndrome coronavirus 2
COVID‐19	Coronavirus disease 19
SARS	Severe acute respiratory syndrome
HGB	Hemoglobin
RBC	Red blood cell count
WBC	White blood cell count
NEU	Neutrophil
LYM	Lymphocyte
MON	Monocyte
EOS	Eosinophil
BAS	Basophil
TP	Total protein
LB	Albumin
GOLB	Globulin
CRP	C‐reactive protein
IgG	Immunoglobulin G
PCT	Procalcitonin
IL‐2	Interleukin‐2
IL‐4	Interleukin‐4
IL‐6	Interleukin‐6
IL‐10	Interleukin‐10
IL‐17	Interleukin‐17
IFN	Interferon
TNF	Tumor necrosis factor

## 3. Discussion

In December 2019, a novel strain of coronavirus, SARS‐CoV‐2, emerged and rapidly spread worldwide, leading to the COVID‐19 pandemic [5]. SARS‐CoV‐2 is an RNA virus that is mainly transmitted through person‐to‐person contact [6]. Studies have confirmed that the pathophysiology of severe SARS‐CoV‐2 infection is largely driven by excessive activation of cytokines, free radicals, and chemokines. Cytokine release syndrome (CRS) can lead to ARDS and is considered a major cause of mortality in patients with severe SARS‐CoV‐2 infection [7].

MM can damage the body’s immune system, including B cells, T cells, natural killer cells, dendritic cells, and the complement system. This can lead to severe immunosuppression and significantly increases the risk of infection, which may occur in some patients even before the initiation of MM treatment [8]. Due to the advanced age, comorbidities, and disease characteristics of patients with MM, as well as the impacts of high‐dose chemotherapy, targeted therapies, immunosuppressive treatments, autologous stem cell transplantation (ASCT), and novel cell‐based therapies, both humoral and cellular immunity are markedly impaired. Consequently, T‐cell and B‐cell functions remain incompletely restored, thereby increasing the risk of severe COVID‐19 infection [9–14]. This immunosuppressed condition may progress to ARDS and thrombotic complications, both of which are associated with increased mortality rates. Severe infection with COVID‐19 is a major risk factor for mortality in patients with MM [15]. A study evaluating the clinical characteristics of 167 patients with MM and 167 noncancer patients infected with COVID‐19 found that 77 patients with MM progressed to moderate‐to‐severe disease, and 8% developed critical illness. Compared to noncancer patients, patients with MM had a significantly higher need for supplemental oxygen, and a greater proportion required noninvasive or invasive ventilation. Despite receiving combination therapies, the mortality rate among some patients with MM remained as high as 34% [15]. For symptomatic COVID‐19 patients with MM, it is recommended to temporarily suspend MM treatment. Oral antiviral agents such as nirmatrelvir/ritonavir and molnupiravir, as well as remdesivir, have demonstrated efficacy against the Omicron variant. These treatments should be initiated upon confirmation of COVID‐19 infection or within 5 days of symptom onset [16]. Krejci M et al. conducted a retrospective analysis involving 50 patients with MM diagnosed with COVID‐19 who received various novel therapies. Among them, 12 patients were treated with antiviral agents combined with convalescent plasma, nine recovered, while three died [4]. Another study further confirmed the effectiveness of nirmatrelvir/ritonavir in treating SARS‐CoV‐2 infection among patients with hematological malignancies, contributing to reduced mortality [17].

The male patient in this study was newly diagnosed with MM and developed fever, cough, and sputum production during treatment, which are symptoms commonly associated with COVID‐19 infection [18]. Blood routine results indicated a significant increase in the number of lymphocytes during COVID‐19 infection, while cytokine analysis revealed a significant increase in IL‐6 levels, which subsequently caused systemic inflammation. The patient showed obvious symptoms of respiratory distress, which was consistent with findings reported in the literature. Chest CT changes also confirmed the inflammatory process. Subsequently, bronchoscopy was performed, and the bronchoalveolar lavage fluid was submitted for SARS‐CoV‐2 detection via NGS. The clinical condition of the patient gradually improved after treatment with nirmatrelvir, indicating that nirmatrelvir was effective in the treatment of novel coronavirus infection. BAL–NGS confirmation in a hypoxemic, immunocompromised host prompted early nirmatrelvir/ritonavir. Concurrently, IL‐6 levels decreased significantly to the normal range. It has been reported that IL‐6 plays a crucial role in the pathogenesis of MM. Tocilizumab, an inhibitor targeting IL‐6 signaling, is very effective in inhibiting MM cell proliferation and may serve as a promising future immunotherapy option for MM. However, in this study, tocilizumab was not administered due to financial constraints, which is a limitation of the case.

## 4. Conclusion

MM is a hematologic malignancy frequently characterized by profound immune dysfunction, leading to a high risk of infection and infection‐related mortality. Individuals with MM have been among the most severely affected populations during the COVID‐19 pandemic. These patients exhibit an increased susceptibility to severe disease and death following SARS‐CoV‐2 infection, which may be due to impaired immune responses against SARS‐CoV‐2 or reduced efficacy of COVID‐19 vaccines. Additionally, overlapping pathophysiological mechanisms, such as endothelial damage in both MM and COVID‐19, may further contribute to their heightened vulnerability. Given these intersecting risks, the importance of timely antiviral therapy and appropriate symptomatic and supportive care is particularly emphasized.

The patient enrolled in this study was in a critical condition due to a severe pulmonary infection, which posed an ongoing life‐threatening risk. Given the inability of throat swabs to reliably detect the pathogen, diagnostic and therapeutic interventions presented significant challenges. After thorough and repeated discussions with the patient’s family regarding the clinical status and proposed procedures, electronic bronchoscopy combined with bronchoalveolar lavage under local anesthesia was performed. Subsequent NGS and pathological analyses confirmed a novel coronavirus infection, with histopathological findings indicative of inflammatory changes. Following antiviral therapy and comprehensive supportive care, the patient’s clinical symptoms showed marked improvement. Notably, during the bronchoscopy procedure, the patient experienced sudden cardiac and respiratory arrest, necessitating immediate resuscitation. Thanks to the coordinated efforts of the medical team and full cooperation from the family, the patient was successfully stabilized. This case holds significant clinical value in accumulating evidence‐based experience for the management of critically ill patients with similar presentations in future practice.

## Funding

No funding was received for this manuscript.

## Conflicts of Interest

The authors declare no conflicts of interest.

## Data Availability

The data that support the findings of this study are available in the supporting information of this article.
